# Supplementary material for: Post-meiotic mechanism of facultative parthenogenesis in gonochoristic whiptail lizard species
Source: eLife. 2024 Jun 7;13:e97035. doi: 10.7554/eLife.97035 (PMC11161175; doi:10.7554/eLife.97035)
Supplement: Supplementary file 1. [file elife-97035-supp1.docx]

**Supplementary file 1.** *A. marmoratus* genome assembly statistics

| **Statistic** | **Meraculous** | **HiRise** | **Units** |
| --- | --- | --- | --- |
| N50 | 1600688 | 32220929 | bp |
| N90 | 413554 | 7979424 | bp |
| max_scaffold | 11507399 | 85027298 | bp |
| mean_scaffold | 188287.18 | 428523.466 | bp |
| median_scaffold | 1703 | 1450 | bp |
| min_scaffold | 1000 | 927 | bp |
| number of scaffolds | 8705 | 3826 | bp |
| perc_A | 27.06 | 27.04 | % |
| perc_C | 20.14 | 20.12 | % |
| perc_G | 20.13 | 20.13 | % |
| perc_N | 5.64 | 5.67 | % |
| perc_T | 27.04 | 27.04 | % |
| perc_genome_covered_by_scaffolds_greater_than_100kb | 97.99 | 99.46 | % |
| perc_genome_covered_by_scaffolds_greater_than_10kb | 99.28 | 99.63 | % |
| perc_genome_covered_by_scaffolds_greater_than_1mb | 69.83 | 98.47 | % |
| scaffolds_greater_than_100Mb | 0 | 0 |  |
| scaffolds_greater_than_100kb | 1588 | 133 |  |
| scaffolds_greater_than_10Mb | 2 | 45 |  |
| scaffolds_greater_than_10kb | 2076 | 223 |  |
| scaffolds_greater_than_1Mb | 545 | 90 |  |
| total bases | 1639039918 | 1639530780 | bp |
| non_n_GC% | 42.67 | 42.67 | % |
| non_n_AT% | 57.33 | 57.33 | % |
