# Supplementary material for: Post-meiotic mechanism of facultative parthenogenesis in gonochoristic whiptail lizard species
Source: eLife. 2024 Jun 7;13:e97035. doi: 10.7554/eLife.97035 (PMC11161175; doi:10.7554/eLife.97035)
Supplement: Supplementary file 2. [file elife-97035-supp2.docx]

**Supplementary file 2.** Trinity assembly statistics

|  | **Trinity initial assembly** | **Final filtered Trinity assembly** |
| --- | --- | --- |
| Mean contig (bp) | 1146.41188 | 1332.98712 |
| Median contig (bp) | 505 | 686 |
| Min contig (bp) | 224 | 174 |
| Max contig (bp) | 24454 | 24454 |
| Number of contigs | 513013 | 119728 |
| N50 (bp) | 2415 | 2490 |
| N90 (bp) | 393 | 512 |
| Total bases (bp) | 588124198 | 159595882 |
