# Supplementary material for: Post-meiotic mechanism of facultative parthenogenesis in gonochoristic whiptail lizard species
Source: eLife. 2024 Jun 7;13:e97035. doi: 10.7554/eLife.97035 (PMC11161175; doi:10.7554/eLife.97035)
Supplement: Supplementary file 3. [file elife-97035-supp3.docx]

**Supplementary file 3.** MAKER2 summary

| **Feature** | **Source** | **Count** |
| --- | --- | --- |
| CDS | MAKER2 | 222450 |
| exon | MAKER2 | 164669 |
| five_prime_UTR | MAKER2 | 22402 |
| gene | MAKER2 | 25856 |
| intron | MAKER2 | 189732 |
| mRNA | MAKER2 | 44461 |
| three_prime_UTR | MAKER2 | 21729 |
