# Supplementary material for: Post-meiotic mechanism of facultative parthenogenesis in gonochoristic whiptail lizard species
Source: eLife. 2024 Jun 7;13:e97035. doi: 10.7554/eLife.97035 (PMC11161175; doi:10.7554/eLife.97035)
Supplement: Supplementary file 4. [file elife-97035-supp4.docx]

**Supplementary file 4.** Information for all *A. marmoratus* animals sequenced

| **ID** | **Analysis type** | **Library type** | **Sex** | **Tissue** | **Sequencing** | **Number of read pairs** | **Read length** |
| --- | --- | --- | --- | --- | --- | --- | --- |
| 8450 | Genome assembly | 40 kb mate pair BfaI | Female | Liver | Illumina MiSeq | 7325967 | 251 |
| 8450 | Genome assembly | 40 kb mate pair CVIQI | Female | Liver | Illumina MiSeq | 7578268 | 251 |
| 8450 | Genome assembly | 5 kb mate pair | Female | Liver | Illumina HiSeq 2500 | 68124235 | 151 |
| 8450 | Genome assembly | 8 kb mate pair | Female | Liver | Illumina HiSeq 2500 | 62138640 | 151 |
| 8450 | Genome assembly | 2-15 kb mate pair | Female | Liver | Illumina HiSeq 2500 | 67949265 | 151 |
| 8450 | Genome assembly | Chicago library | Female | Liver | Illumina HiSeq 2500 | 52923610 | 101 |
| 8450 | Genome assembly | Chicago library | Female | Liver | Illumina HiSeq 2500 | 207139609 | 101 |
| 8450 | Genome assembly | Paired-end | Female | Liver | Illumina HiSeq 2500 | 376374707 | 251 |
| S30700 | Full genome sequencing | Paired-end | Female | Tail | Illumina HiSeq 2500 | 106787117 | 151 |
| 9177 | Full genome sequencing | Paired-end | Female | Tail | Illumina HiSeq 2500 | 113372074 | 151 |
| 6993 | Full genome sequencing | Paired-end | Female | Tail | Illumina HiSeq 2500 | 100116690 | 151 |
| 12512 | Full genome sequencing | Paired-end | Female | Tail | Illumina HiSeq 2500 | 102760203 | 151 |
| 12513 | Full genome sequencing | Paired-end | Female | Tail | Illumina HiSeq 2500 | 103885909 | 151 |
| 9721 | Full genome sequencing | Paired-end | Female | Tail | Illumina HiSeq 2500 | 101265101 | 151 |
| 122 | Full genome sequencing | Paired-end | Female | Tail | Illumina HiSeq 2500 | 321903175 | 151 |
| 001 | Full genome sequencing | Paired-end | Male | Liver | Illumina HiSeq 2500 | 96536963 | 151 |
| 003 | Full genome sequencing | Paired-end | Male | Liver | Illumina HiSeq 2500 | 108407900 | 151 |
| 8450 | Full genome sequencing | Paired-end | Female | Liver | Illumina HiSeq 2500 | 103851051 | 151 |
| 11225 | Transcriptome assembly | Poly A stranded RNA-seq | Male | Blood | Illumina HiSeq 2500 | 260779309 | 101 |
| S237-2 | Transcriptome assembly | Poly A stranded RNA-seq | Unkown | Embryo | Illumina HiSeq 2500 | 253832665 | 101 |
