# Supplementary material for: Post-meiotic mechanism of facultative parthenogenesis in gonochoristic whiptail lizard species
Source: eLife. 2024 Jun 7;13:e97035. doi: 10.7554/eLife.97035 (PMC11161175; doi:10.7554/eLife.97035)
Supplement: Supplementary file 5. [file elife-97035-supp5.docx]

**Supplementary file 5.** Whole genome sequencing alignment statistics

| **Animal ID** | **12512** | **12513** | **S30700** | **6993** | **9177** | **9721** | **001** | **003** | **122** | **8450** |
| --- | --- | --- | --- | --- | --- | --- | --- | --- | --- | --- |
| **reads total** | 2.1E+08 | 2.12E+08 | 2.18E+08 | 2.04E+08 | 2.32E+08 | 2.07E+08 | 1.97E+08 | 2.21E+08 | 6.56E+08 | 2.12E+08 |
| **secondary** | 4371764 | 4293355 | 4427220 | 4120929 | 4761643 | 4246124 | 3932037 | 4507406 | 12408444 | 4081669 |
| **supplementary** | 0 | 0 | 0 | 0 | 0 | 0 | 0 | 0 | 0 | 0 |
| **duplicates** | 1829281 | 1841233 | 1848550 | 2314935 | 2169035 | 2151550 | 1523198 | 1283723 | 14760637 | 1368644 |
| **mapped** | 2.07E+08 | 2.09E+08 | 2.15E+08 | 2.01E+08 | 2.29E+08 | 2.04E+08 | 1.93E+08 | 2.18E+08 | 6.5E+08 | 2.08E+08 |
| **paired in sequencing** | 2.06E+08 | 2.08E+08 | 2.14E+08 | 2E+08 | 2.27E+08 | 2.03E+08 | 1.93E+08 | 2.17E+08 | 6.44E+08 | 2.08E+08 |
| **read1** | 1.03E+08 | 1.04E+08 | 1.07E+08 | 1E+08 | 1.13E+08 | 1.01E+08 | 96536963 | 1.08E+08 | 3.22E+08 | 1.04E+08 |
| **read2** | 1.03E+08 | 1.04E+08 | 1.07E+08 | 1E+08 | 1.13E+08 | 1.01E+08 | 96536963 | 1.08E+08 | 3.22E+08 | 1.04E+08 |
| **properly paired** | 1.88E+08 | 1.9E+08 | 1.95E+08 | 1.82E+08 | 2.07E+08 | 1.85E+08 | 1.84E+08 | 2.06E+08 | 5.97E+08 | 1.96E+08 |
| **with itself and mate mapped** | 2.02E+08 | 2.04E+08 | 2.09E+08 | 1.95E+08 | 2.22E+08 | 1.99E+08 | 1.88E+08 | 2.13E+08 | 6.36E+08 | 2.02E+08 |
| **singletons** | 1212491 | 1504914 | 1467678 | 1679077 | 1523933 | 1408129 | 1072182 | 1099052 | 1912936 | 993280 |
| **with mate mapped to a different chr** | 12512434 | 12380462 | 12772150 | 11827242 | 13772076 | 12316058 | 3751076 | 5431708 | 34786180 | 5413508 |
| **with mate mapped to a different chr (mapQ>=5)** | 7450173 | 7412852 | 7583394 | 7060453 | 8164436 | 7358325 | 2149235 | 3193570 | 21253145 | 3196901 |
