# Supplementary material for: Post-meiotic mechanism of facultative parthenogenesis in gonochoristic whiptail lizard species
Source: eLife. 2024 Jun 7;13:e97035. doi: 10.7554/eLife.97035 (PMC11161175; doi:10.7554/eLife.97035)
Supplement: Supplementary file 6. [file elife-97035-supp6.docx]

**Supplementary file 6.** Animals confirmed by microsatellite analysis to be of FP origin

| **Species** | **Animal ID** | **Clutch ID** | **Laid-Begin** | **Laid-End** | **Hatch** | **Notes** |
| --- | --- | --- | --- | --- | --- | --- |
| *A. marmoratus* | 6993 | X48 | 21-Oct-07 | 22-Oct-07 |  | Animal was cut from egg, alive, with brain exposed from skull |
| *A. marmoratus* | 8377 | J68 | 21-Oct-08 | 25-Oct-08 |  |  |
| *A. marmoratus* | 8394 | P68 | 29-Oct-08 | 2-Nov-08 |  | Missing a leg, organs exposed at abdomen, face abnormalities, and hunched back |
| *A. marmoratus* | 8449 | M69 | 18-Nov-08 | 22-Nov-08 | 25-Jan-09 | Appeared to have balance issues; hatched with substance still attached to abdomen |
| *A. marmoratus* | 8450 | M69 | 18-Nov-08 | 22-Nov-08 | 25-Jan-09 | Appeared to have balance issues |
| *A. arizonae* | 8677 | K72 | 7-Feb-09 | 10-Feb-09 |  |  |
| *A. marmoratus* | 9070 | V78 | 12-Jun-09 | 16-Jun-09 |  |  |
| *A. marmoratus* | 9177 | F80 | 30-Jul-09 | 3-Aug-09 | 10-Oct-09 |  |
| *A. marmoratus* | 12512 | L131 | 26-Apr-12 | 30-Apr-12 | 8-Jul-12 | Deformed jaw; missing left eye |
| *A. marmoratus* | 12513 | L131 | 26-Apr-12 | 30-Apr-12 | 8-Jul-12 | Deformed jaw, missing right eye |
| *A. arizonae* | 16215 | B187 | 7-Mar-14 | 11-Mar-14 | 2-May-14 | Right side of skull seems to have stunted growth compared to left side, lower mandible protrudes out slightly, right eye seems slightly smaller than left |
| *A. arizonae* | 16216 | B187 | 7-Mar-14 | 11-Mar-14 | 2-May-14 | Missing left eye, malformed jaw, shortened torso |
| *A. marmoratus* | 19687 | T237 | 19-May-15 | 23-May-15 | 1-Aug-15 |  |
| *A. marmoratus* | 19688 | T237 | 19-May-15 | 23-May-15 |  | Multiple craniofacial deformities |
| *A. arizonae* | 23304 | Q288 | 11-Sep-16 | 15-Sep-16 | 15-Nov-16 |  |
| *A. arizonae* | 23507 | D291 | 11-Oct-16 | 15-Oct-16 | 15-Dec-16 |  |
| *A. marmoratus* | 24514 | L303 | 12-Jan-17 | 16-Jan-17 |  |  |
| *A. arizonae* | 25339 | I313 | 3-Mar-17 | 7-Mar-17 |  | Deformed snout, missing left eye |
| *A. marmoratus* | 25384 | R313 | 5-Mar-17 | 9-Mar-17 | 6-Jun-17 |  |
| *A. marmoratus* | 25385 | R313 | 5-Mar-17 | 9-Mar-17 | 6-Jun-17 | Found partially emerged from egg, barely moving, had large mass of egg yolk still attached |
| *A. arizonae* | 29606 | E364 | 27-Apr-18 | 1-May-18 | 3-Jul-18 |  |
| *A. arizonae* | 29607 | E364 | 27-Apr-18 | 1-May-18 | 3-Jul-18 | Congenital shortened torso; hatched alive and died immediately |
| *A. marmoratus* | 30050 | A369 | 29-Jun-18 | 3-Jul-18 |  | No eyes |
