# Supplementary material for: Post-meiotic mechanism of facultative parthenogenesis in gonochoristic whiptail lizard species
Source: eLife. 2024 Jun 7;13:e97035. doi: 10.7554/eLife.97035 (PMC11161175; doi:10.7554/eLife.97035)
Supplement: Supplementary file 7. [file elife-97035-supp7.docx]

**Supplementary file 7.** Microsatellite primer information

| **Microsatellite** | **Oligo 1 sequence (5’ → 3’)** | **Oligo 2 sequence (5’ → 3’)** |
| --- | --- | --- |
| MS1 | TGCATGATGGAGGAATCTTC | CTAGTGGTGATAGAAACATGG |
| MS2 | GATCCTGTTGCCGTTTGGAC | ATGCTTCTAGATGAACCCAC |
| MS3 | ﻿CTCTCCTTGCAAACAGCAGG | ﻿ACATGGGTGAGTTGAGGGTG |
| MS5 | ﻿TATCTCCTGGCTATCAAGAC | ﻿TTAGTTAGCCCTTAGCACAG |
| MS6 | CACACCCATATTATAAGTGG | CATTCAGATGAAACCTAACC |
| MS7 | AACTAAGTGCTAAGTGTGAC | ACAGTCTTAGAGATCACAAG |
| MS8 | ACACCCAAAGTCCTCAACAG | CTAGTACATGTGTAAGGGTG |
| MS9 | GCAGACTCATAGTTGAAGTG | TCTGGGAATACCAGGTTCAC |
| MS10 | GACCAATAATGTGGAAGCTG | ACATGGCTGAGTAATTGGTG |
| MS12 | TACCCACCTGGAGATGTTTAG | AGGACGCCTTAAAATAGGAAG |
| MS14 | TGGAGGCAGTCTTGGTATC | GAACATTGACCGCATCAC |
| MS15 | TTAAAGCAGAGGTCAGGTTATC | GATGGAAGAATAGGATGATGAA |
| MS16 | TTTAATGCATCCACTGAGTC | GGAATATAGTGGCATATCAG |
| MS17 | AATCCTGAACCTACGGTAAGC | TGCCAGAAAATAGAGGGAAG |
| MS18 | AATTAATGTGCAGCACTAT | GGCAGTTTTTCAGCTAAG |
| MS19 | AAAAAGAAAAGGAAGAACTAA | TGAGACAAGTTGGGTAGA |
